# Real-World Evaluation of the Interaction Between Rifampicin and Warfarin: A Retrospective Observational Study

**DOI:** 10.1155/adpp/5607280

**Published:** 2025-07-29

**Authors:** Lina Naseralallah, Dima Nasrallah, Somaya Koraysh, Ahmad R. Al-Qudimat

**Affiliations:** ^1^Pharmacy Department, Hamad Medical Corporation, Doha, Qatar; ^2^College of Medicine, QU Health, Qatar University, Doha, Qatar; ^3^Surgery Research Department, Hamad Medical Corporation, Doha, Qatar; ^4^College of Health Sciences, QU Health, Qatar University, Doha, Qatar

**Keywords:** drug interaction, international normalized ratio, pharmacoepidemiology, rifampicin, warfarin

## Abstract

**Background:** Drug–drug interactions (DDIs) pose a significant challenge in drug therapy, particularly due to concerns about the safety and effectiveness of combined medications. Rifampicin is a strong inducer of the enzyme CYP2C9, which likely reduces warfarin's effectiveness. This study aims to investigate the prevalence and severity of clinically significant interactions by analyzing changes in international normalized ratio (INR) levels.

**Method:** The study was a retrospective observational analysis conducted from 2014 to 2024, using data on INR measurements from patients treated concurrently with warfarin and rifampicin. INR values were recorded at multiple time points, including baseline, during concomitant treatment, and after discontinuation.

**Results:** A significant proportion (86.3%) of the 102 patients using warfarin concurrently with rifampicin experienced a DDI, resulting in a notable reduction in INR (*p* < 0.0001), with a median decrease to 1.3 (IQR 1.1–1.6). Among the patients who achieved the target INR (55.9%), warfarin dose was increased by a median of 5.5 mg, and the median time to INR stabilization was 18 days. However, approximately 31% of patients did not reach the target INR despite dose adjustments.

**Conclusion:** The warfarin–rifampicin interaction is clinically significant, as it can diminish warfarin's anticoagulant effect, potentially compromising patient health outcomes. Close monitoring and individualized treatment plans are crucial for patients receiving both medications concurrently.

**Summary**

• Drug interactions can make it difficult to use medications safely and effectively.
• This study looked at how rifampicin, a drug used to treat infections like tuberculosis, interacts with warfarin, a common blood thinner.
• Rifampicin speeds up how the body processes certain medications, which reduces warfarin's ability to thin the blood, increasing the risk of dangerous blood clots.
• We reviewed the medical records of 102 patients who used both drugs between 2014 and 2024.
• The findings showed that most patients (86.3%) experienced a drop in their blood-thinning levels, measured by the international normalized ratio (INR)—a test that indicates how long it takes blood to clot and helps ensure blood thinners are working safely.
• The median INR dropped to 1.3, which is below the safe range of 2-3.
• Some patients needed a higher dose of warfarin to bring their INR back to normal, but 31% never reached the safe levels even after adjusting their dose.
• The study concluded that this drug interaction is common and serious, requiring close monitoring.
• Doctors must tailor treatment for each patient to ensure that the blood thinner works effectively.

## 1. Introduction

Drug–drug interactions (DDIs) present a significant challenge in drug therapy due to concerns related to the safety or efficacy of either or all of the coadministered medications [1]. DDIs vary on the scale of clinical relevance and importance. Some DDIs might be associated with minor or no significant adverse drug reactions (ADRs) and even in some cases could lead to beneficial, synergistic effects [2]. The risk of these specific DDIs tend to be overrated in the literature and different electronic drug interaction screening software [3]. Nevertheless, some DDIs can result in serious ADRs; a retrospective study assessing ADRs reports over a 10-year period estimated that DDIs were responsible for over 30% of all ADRs [4]. DDIs have also been linked to avoidable patient harm including treatment failure, drug-related morbidity, emergency department visits, hospital admission, prolonged hospital stays, and even death [5–10].

Warfarin is a vitamin K antagonist used for the treatment and prevention of thromboembolic disorders [11]. It is a narrow therapeutic index drug, requiring frequent monitoring of the international normalized ratio (INR) to balance efficacy and safety, especially when initiating or stopping concomitant medications [12]. Warfarin remains the preferred oral anticoagulation treatment for valvular heart disease and atrial fibrillation, as well as venous thromboembolism (VTE) at unusual sites [13, 14]. The drug is a racemic mixture of 2 optically active isomers, though the S-enantiomer is roughly 5 times more potent than the R-enantiomer [15]. The more potent warfarin S-isomer is extensively metabolized by the cytochrome P450 (CYP) 2C9 liver enzyme which makes it highly susceptible to DDIs.

Given CYP2C9 metabolizes warfarin, inducers of this enzyme are believed to diminish its anticoagulant effect [15]. Rifampicin is known to be a potent inducer of CYP2C9, suggesting that it lowers the effectiveness of warfarin [16]. Rifampicin is a frontline anti-infective agent used in the treatment of tuberculosis, leprosy, and other bacterial infections [17]. Several case reports and series involving patients receiving both drugs concurrently have demonstrated that rifampicin increases warfarin dose requirements—sometimes up to 20 mg—and in some cases, the target INR remains unattained despite dose escalation [18–22]. Although formal clinical guidelines outlining specific management protocols are lacking, resources such as the British National Formulary (BNF) and the American College of Chest Physicians (ACCP) guidelines recommend close INR monitoring and individualized warfarin dose adjustment when coadministered with rifampicin, due to the high risk of subtherapeutic anticoagulation [23, 24].

Given the previously reported cases, the prolonged treatment courses of both warfarin and rifampicin—often unavoidable due to limited alternative therapies—and the critical impact of suboptimal anticoagulation, we conducted a retrospective cohort study to evaluate the prevalence and severity of clinically significant interactions, as measured by changes in INR. Although the interaction between these two drugs is well-documented, there is a lack of evidence from routine clinical settings. Consequently, this study holds significant importance as it provides real-world data spanning a decade. The findings aim to guide clinicians on what to expect when coprescribing these medications and inform appropriate monitoring and follow-up schedules. This approach will help ensure the safe initiation and discontinuation of rifampicin in warfarin-treated patients, ultimately improving clinical outcomes.

## 2. Methods

### 2.1. Ethics Approval

The Medical Research Center (MRC) at Hamad Medical Corporation (HMC) approved this study (*reference number: MRC-01-23-342*). This was a secondary data analysis of anonymized patient data; hence, the requirement for written informed consent was waived.

### 2.2. Study Design and Settings

We conducted a retrospective analysis of patients' medical records who used rifampicin and warfarin concomitantly at any of the 14 hospitals under HMC between April 2014 and February 2024. The study was reported as per the STrengthening the Reporting of OBservational studies in Epidemiology (STROBE) statement for observational studies [25].

### 2.3. Participants and Cohort Identification

The study cohort comprised adult patients (aged ≥ 18 years) who were taking warfarin and rifampicin simultaneously and had documented INR levels at baseline and during follow-up. We excluded patients who received warfarin or rifampicin for less than 14 days, as well as patients who did not have documented follow-up INR levels (i.e., less than 2 INR readings separated by at least 3 days and occurred after more than 14 days from rifampicin initiation). This is because in most cases, the onset of the interaction effect between rifampicin and warfarin appears after more than 14 days [19, 20, 26, 27]. Patients receiving other concomitant medications known to strongly induce or inhibit CYP2C9 or otherwise significantly interact with warfarin (such as carbamazepine, phenytoin, amiodarone, or fluconazole) were excluded to minimize confounding effects on INR values. This approach helped isolate the impact of rifampicin on warfarin anticoagulation.

To identify our cohort, two different sheets were generated through a computer-based pharmacy system for patients who took warfarin and rifampicin in the inpatient and outpatient settings (warfarin clinic). To ensure data quality, automated cross-checking between the two sheets was done by an information technology pharmacist to preclude any overlap and generate a single sheet of all the patients who took both medications at the same time. To further ensure patients' eligibility for inclusion in this study, a manual check was done by one of the researchers for each identified patient. A final sheet of all the patients was generated and used in this study.

### 2.4. Outcomes

The outcome measures were as follows:

• The prevalence of clinically significant DDI among patients on warfarin and rifampicin. A clinically significant DDI refers to an interaction between warfarin and rifampicin that produces a measurable change in patient outcomes, such as a significant alteration in INR values, which potentially compromises the safety or effectiveness of therapy. This type of interaction necessitates a clinical intervention, including warfarin dose adjustment, intensified INR monitoring, or modification/discontinuation of one or both drugs [28].
• The change in INR value from baseline after initiation of warfarin or rifampicin.
• The change in warfarin dose to achieve the target INR value.
• Time to INR stabilization.
• The proportion of patients who achieved the target INR level.

### 2.5. Data Collection

The following data were obtained for included patients: sociodemographic (age, sex, and ethnicity); rifampicin indication, dose, and duration; warfarin dose at baseline, upon reaching the INR target, and after stopping rifampicin; INR value at baseline and throughout rifampicin treatment; and patient management if INR target was not achieved.

### 2.6. Data Handling and Analysis

We graphically depicted changes in INR values by mapping median, interquartile, and 10th and 90th percentiles during the study window. To formally assess whether the mean INR significantly decreased following rifampicin initiation, we compared the latest INR result before initiating rifampicin with the first INR result after 14 days of rifampicin initiation using a paired *t*-test. In addition, we evaluated median INR changes for all rifampicin-treated patients and by different dose groups in secondary analyses. Finally, we calculated the proportion of patients with INR values below the therapeutic range (INR < 2 and < 1) by comparing the periods 1–4 weeks before and 2–6 weeks after rifampicin initiation. All data cleaning and analyses were conducted using the statistical package IBM SPSS Version 22 software.

## 3. Results

The study included 102 participants with a mean age of 50.8 (±15.9) years, who were mostly male (*n* = 81, 79.4%) and Asian (*n* = 58, 56.9%). Tuberculosis (63.7%) was the main indication for rifampicin use, followed by endocarditis (29.4%), at lesser frequency osteomyelitis (2.9%) and bacteremia (2.0%); daily dose regimen of 600 mg was given in most cases (73.5%). Around 56% of patients received warfarin prior to starting rifampicin, while the remaining 43% began warfarin either concurrently with or after starting rifampicin (Table 1).

**Figure 1:**
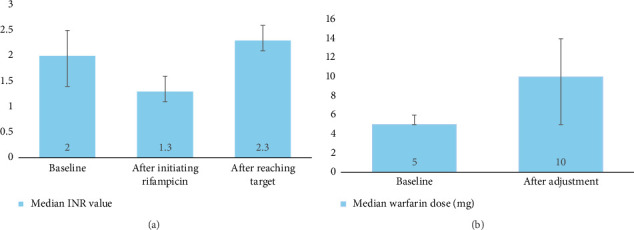
(a) Median INR value at baseline, after initiating rifampicin, and after reaching target INR. (b) Median warfarin dose before and after rifampicin initiation in patients who achieved target INR. Target INR range is considered to be between 2.0 and 3.0.

**Figure 2:**
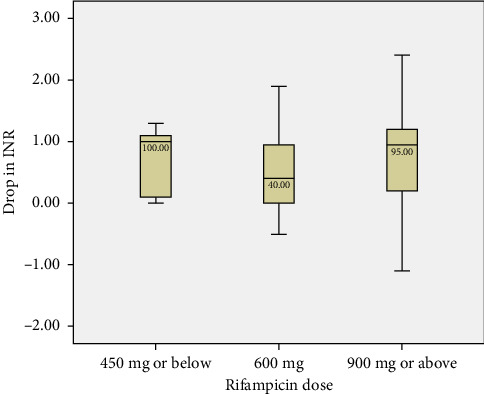
Box and whisker plot showing median change in INR among patients treated with rifampicin, stratified by dose: ≤ 450 mg, 600 mg, and ≥ 900 mg. The box represents the interquartile range (25th–75th percentiles), the line within the box indicates the median, and the whiskers denote the 10th and 90th percentiles. Therapeutic INR range is considered to be between 2.0 and 3.0.

At baseline, the median warfarin dose was 5 mg, with an interquartile range (IQR) of 5–6 mg, indicating a relatively narrow variation in dosage among patients. The median INR at baseline was 2.0 (IQR 1.4–2.5), reflecting a stable therapeutic range before the introduction of rifampicin (Figure 1(a)). Following the introduction of rifampicin, a significant drop in INR levels was observed (*p* < 0.0001), with the median INR decreasing to 1.3 (IQR 1.1–1.6). No significant difference was noted in the reduction of INR across the various dosage regimens (450 mg or below, 600 mg, and 900 mg or above) (Figure 2). In response, the median warfarin dose was increased to 10 mg (IQR 5–14) in an effort to stabilize INR levels. After these adjustments, the target INR was reached, with a median value of 2.3 (IQR 2.1–2.6) (Figure 1(b)).

Of the 102 patients included in the study, 57 (55.9%) achieved the target INR. Among these patients, the median INR nadir was 1.3 (IQR 1.2–1.5), indicating the lowest recorded value, with a median drop in INR of 0.5 (IQR 0.1–1.0) from baseline. To achieve stabilization, warfarin dose was increased by a median of 5.5 mg (IQR 1.5–8.5), and the time required for INR stabilization was 18 days (IQR 10–24). In contrast, 31 patients (30.4%) did not reach the target INR, while 14 patients (13.7%) maintained stable INR values throughout rifampicin therapy without significant dose adjustments (Table 2). These patients did not experience either subtherapeutic or supratherapeutic INR excursions.

The management of the interaction involved increasing the warfarin dose for 68 patients (66.7%). Additionally, 6 patients (5.9%) were shifted to parenteral anticoagulation, and 1 patient (0.9%) was switched to a direct oral anticoagulant (DOAC).

## 4. Discussion

The study involved 102 participants, with a mean age of 50.8 years, predominantly male (79.4%) and Asian (56.9%). The baseline median warfarin dose was 5 mg, and the median INR was 2.0, indicating stable therapeutic levels. However, following the introduction of rifampicin, a significant decrease in INR was observed (*p* < 0.0001), with the median dropping to 1.3. No significant differences were noted across the various rifampicin dosage regimens. This prompted an increase in the median warfarin dose to 10 mg, resulting in a subsequent median INR of 2.3. Of the participants, 57 (55.9%) reached the target INR, with a median nadir of 1.3 and a median drop of 0.5 from baseline. Stabilization required a median warfarin dose increase of 5.5 mg and took a median of 18 days. Conversely, 31 patients (30.4%) did not achieve the target INR, while 14 (13.7%) maintained stable values throughout treatment without significant fluctuations. Importantly, this stable group is distinct from those who developed supratherapeutic INR levels (e.g., INR > 4), which were primarily observed after rifampicin discontinuation. The predominant management strategy was to increase the warfarin dose for 68 patients (66.7%).

Although several case reports and case series have discussed the DDI between rifampicin and warfarin, as well as the management techniques adopted [18–22], to our knowledge, this is the first study to estimate the prevalence of clinically significant interactions among patients on concomitant therapy. Identifying such interactions helps prioritize patient safety, streamline care, and ensure that healthcare interventions are both meaningful and necessary. Our findings revealed that such interactions are highly prevalent, occurring in up to 86.3% of the population. This demands clinicians' attention and surveillance, as the reduction in warfarin's anticoagulant activity could potentially lead to subtherapeutic INR levels and an increased risk of thromboembolic events, such as stroke or deep vein thrombosis [29].

Consistent with previous case reports that indicated warfarin dose increases of up to 3–5 times the pre-rifampicin dose, our findings demonstrated that this pharmacokinetic interaction requires substantial dose adjustments to maintain therapeutic INR levels [30, 31]. However, in our cohort, the median warfarin dose increased approximately 2-fold—from a baseline median of 5 mg to 10 mg (IQR 5–14)—which is somewhat lower than previously reported. This discrepancy may be attributed to the lower initial warfarin doses among patients in our study, differences in patient populations, or clinical management practices. Notably, even higher doses of up to 20 mg per day have been documented in the literature [18].

It should also be emphasized that although approximately 56% of patients achieved the target INR, around one-third did not, highlighting the variability in patient response to warfarin dose adjustments. Similarly, Krajewski reported that patients often struggle to maintain therapeutic INR levels, even with significant warfarin dose increases, due to rifampicin's strong induction of CYP enzymes [32]. These results underscore the need for individualized dose titration and vigilant INR monitoring during rifampicin treatment. This finding should also be interpreted in light of the impact of genetic polymorphisms (e.g., CYP2C9, VKORC1, and CYP4F2) on the unexplained interpatient variability observed in patients receiving both warfarin and rifampicin. A Qatar-based case-control study suggested that carriers of warfarin-sensitizing CYP2C9/VKORC1 genotypes are more likely to achieve target INR levels at modest daily doses, whereas noncarriers may require more extensive dose escalations [16]. It is thus advisable to consider genotyping to identify potential responders to feasible doses and to guide prescribers in determining when larger dose escalations are needed for patients with normal CYP2C9/VKORC1 genotypes. This interpatient variability highlights the potential benefit of integrating early pharmacogenetic testing (e.g., for CYP2C9 and VKORC1 genotypes) into routine clinical care. Such testing could help identify patients likely to require higher or lower warfarin doses when coadministered with rifampicin, allowing more precise dose adjustments and targeted INR monitoring from treatment initiation. Incorporating pharmacogenetic information may reduce the time to achieve stable anticoagulation and minimize the risk of adverse events related to subtherapeutic or supratherapeutic INR levels. Future research should aim to identify patient-specific factors that influence the rifampicin–warfarin interaction and develop tailored dosing protocols to improve clinical outcomes, including evaluating the clinical utility and cost-effectiveness of integrating pharmacogenetic testing into routine practice to optimize anticoagulation outcomes in this high-risk group.

Our findings indicated that after the withdrawal of rifampin, INR levels increased significantly reaching supratherapeutic levels, which suggests that managing warfarin dosing post-rifampicin can also be challenging. A study by Martins et al. reported cases where patients experienced significant hematuria following the discontinuing of rifampicin [33]. Additionally, another case report noted a 50% decrease in warfarin requirement after stopping rifampicin [18]. These observations highlight the necessity for close follow-up even after rifampicin discontinuation to avoid any complications such as bleeding.

Alternative strategies to mitigate the interaction between rifampicin and warfarin have been proposed, including the use of rifabutin; a less potent enzyme inducer. Alnewais et al. suggested that rifabutin could be a viable alternative to rifampicin for patients on warfarin, as it has a weaker effect on CYP2C9 and CYP3A4 and results in less drastic warfarin dose adjustments [34]. However, rifabutin may not always be suitable due to its lower efficacy in treating certain infections, and in our study, none of the patients were switched to rifabutin. Nonetheless, one patient (0.9%) was transitioned to a DOAC due to difficulties in maintaining therapeutic INR.

While warfarin remains the preferred oral anticoagulant for certain indications, particularly in patients with mechanical heart valves or valvular atrial fibrillation, DOACs have gained popularity due to their more predictable pharmacokinetics and fewer DDIs [13, 14]. However, DOACs are not free from interaction risks; rifampicin is known to induce CYP enzymes and P-glycoprotein, potentially reducing DOAC plasma levels and anticoagulant efficacy [35]. This highlights the importance of cautious patient selection and close monitoring when considering DOACs in patients requiring rifampicin therapy. Further studies are needed to evaluate the safety and effectiveness of DOACs in this setting.

### 4.1. Strengths and Limitations

One of the key strengths of this study is its extended follow-up period, spanning a decade (2014–2024). Additionally, the inclusion of a diverse patient population, with participants from various ethnic backgrounds, enhances the generalizability of the findings across different demographic groups. The rigorous monitoring of INR levels and dose adjustments allowed us to generate detailed data on the magnitude of dose changes and the time required to achieve therapeutic INR, which is critical for informing clinical practice.

However, the study also has several limitations that must be acknowledged. As a retrospective design, it is subject to inherent biases, including potential inaccuracies in medical records and incomplete data capture. Furthermore, because the study relied on clinical records, factors such as genetic polymorphisms (CYP2C9/VKORC1) that could have influenced warfarin metabolism and patient responses were not routinely available or assessed. Additionally, the distribution of patients across rifampicin dose groups was unbalanced, with the majority (73.5%) receiving 600 mg daily, while only a small number received ≤ 450 mg (4.9%) or ≥ 900 mg (21.6%). This imbalance may have limited the ability to detect dose-dependent differences in INR changes, particularly in the smallest subgroup. As such, findings related to rifampicin dose stratification should be interpreted with caution.

## 5. Conclusion

The warfarin–rifampicin interaction is clinically significant, as it can diminish warfarin's anticoagulant effect, increasing the risk of subtherapeutic INR levels and, consequently, a higher likelihood of thromboembolic events such as stroke or deep vein thrombosis. The variability in patient response underscores the need for careful monitoring of INR levels and timely dose adjustments to maintain effective anticoagulation. In patients receiving both medications, individualized treatment plans are critical to mitigate the risks associated with this interaction.

## Figures and Tables

**Table 1:** Baseline characteristics of included patients.

		**(*n* = 102)**	

Age (mean, STD)		50.8	15.9

Gender	Male	81	79.4%
	Female	21	20.6%

Nationality	Arab	38	37.3%
	Asian (non-Arab)	58	56.9%
	African (non-Arab)	6	5.9%

Rifampicin indication	Tuberculosis	65	63.7%
	Endocarditis	30	29.4%
	Osteomyelitis	3	2.9%
	Bacteremia	2	2.0%
	Other	2	2.0%

Rifampicin daily dose	450 mg or below	5	4.9%
	600 mg	75	73.5%
	900 mg or above	22	21.6%

Rifampicin treatment duration (days) (median (IQR))		96.5 (30–202)	

Warfarin start time	Before rifampicin	58	56.9%
	With or after rifampicin	44	43.1%

**Table 2:** Warfarin and INR characterization before and after initiating rifampicin.

Before initiation of rifampicin	
Warfarin dose at baseline (median (IQR))	5 (5-6)
Baseline INR (median (IQR))	2 (1.4–2.5)

**After initiation of rifampicin**	

INR level after starting rifampicin (median, IQR))	1.3 (1.1–1.6)
Warfarin dose once INR stabilized (median (IQR))	10 (5–14)
Target INR reached after adjusting warfarin dose (median (IQR)	2.3 (2.1–2.6)
INR reached desired target	
Yes	57 (55.9%)
INR nadir after starting rifampicin^∗^	1.3 (1.2–1.5)
Drop in INR (from baseline)^∗^	0.5 (0.1–1.0)
Change in dose^∗^	5.5 (1.5–8.5)
Time to INR stabilization (days)^∗^	18 (10–24)
No	31 (30.4%)
Remained stable	14 (13.7%)
INR reached 4 or above	14 (13.7%)
Management of the drug–drug interaction	
Warfarin dose increased	68 (66.7%)
No intervention	27 (26.5%)
Shifted to parenteral anticoagulation	6 (5.9%)
Shifted to DOAC	1 (0.9%)

^∗^Median, IQR.

## Data Availability

The data that support the findings of this study are available on request from the corresponding author. The data are not publicly available due to privacy or ethical restrictions.
